# The emerging determinants of replication fork stability

**DOI:** 10.1093/nar/gkab344

**Published:** 2021-05-12

**Authors:** Tanay Thakar, George-Lucian Moldovan

**Affiliations:** Department of Biochemistry and Molecular Biology, The Pennsylvania State University College of Medicine, Hershey, PA 17033, USA; Department of Biochemistry and Molecular Biology, The Pennsylvania State University College of Medicine, Hershey, PA 17033, USA

## Abstract

A universal response to replication stress is replication fork reversal, where the nascent complementary DNA strands are annealed to form a protective four-way junction allowing forks to avert DNA damage while replication stress is resolved. However, reversed forks are in turn susceptible to nucleolytic digestion of the regressed nascent DNA arms and rely on dedicated mechanisms to protect their integrity. The most well studied fork protection mechanism involves the BRCA pathway and its ability to catalyze RAD51 nucleofilament formation on the reversed arms of stalled replication forks. Importantly, the inability to prevent the degradation of reversed forks has emerged as a hallmark of BRCA deficiency and underlies genome instability and chemosensitivity in BRCA-deficient cells. In the past decade, multiple factors underlying fork stability have been discovered. These factors either cooperate with the BRCA pathway, operate independently from it to augment fork stability in its absence, or act as enablers of fork degradation. In this review, we examine these novel determinants of fork stability, explore the emergent conceptual underpinnings underlying fork protection, as well as the impact of fork protection on cellular viability and cancer therapy.

## INTRODUCTION

The genome is at its most vulnerable during DNA replication since this process seldom progresses completely unobstructed. Several factors of endogenous and exogenous origin can pose obstacles to the progression of replication forks resulting in replication stress. A global response upon encountering replication stress involves the remodeling of replication forks to enable the annealing of nascent DNA strands, a process known as replication fork reversal. Fork reversal, intrinsically coupled to an initial fork slowing response, is thought to safeguard fork integrity by granting tolerance to transient replication stress. Mechanistically, the protective effect of fork reversal is thought to manifest in distinct ways: (i) Fork regression, which accompanies fork reversal, allows for the template lesion to be brought back into a double-stranded DNA configuration permissive to excision repair; (ii) The regressed complementary arms of nascent DNA can potentially allow for the bypass of template DNA obstructions by employing the nascent strand of the sister chromatid as a template; (iii) Fork reversal allows forks under stress to assume a dormant configuration until the replication stress can be resolved, thereby protecting them from adverse outcomes of unrestrained progression such as fork breakage. However, in certain genetic backgrounds or under conditions of prolonged fork arrest, reversal can render forks susceptible to nucleolytic resection. Excessive nucleolytic resection (also known as degradation) of reversed forks is often associated with the accumulation of chromosomal abnormalities. Fork degradation is thus a prominent mechanism of genome instability. In addition, fork degradation is considered a major predictor of chemosensitivity and cellular fitness. The best described mechanism ensuring protection against fork degradation involves the BRCA proteins (comprised of the breast cancer susceptibility factors BRCA1 and BRCA2) and components of the Fanconi anemia (FA) tumor suppressor pathway, which are known effectors of homologous recombination (HR) in metazoan cells. These mechanisms rely on stabilizing RAD51 nucleofilaments on the regressed nascent DNA of reversed replication forks. However, recent work has revealed a multitude of additional factors and pathways that directly affect fork protection by either influencing RAD51 nucleofilament formation or through completely distinct mechanisms. In this review, we examine the recent advances in the understanding of the classical fork protection pathways, as well as the emerging determinants of fork protection.

## THE CLASSICAL FA/BRCA FORK PROTECTION PATHWAY

The role of RAD51 in orchestrating protection of stressed replication forks was first documented in *Xenopus laevis* egg extracts. It was observed that upon inhibiting the chromatin binding of RAD51, the nuclease MRE11 drives the accumulation of internal ssDNA gaps in replicating DNA (1). Subsequently, a more general role for RAD51 in protecting nascent DNA was established in mammalian cells when it was revealed that loss of BRCA2, a known effector of the HR pathway, resulted in excessive MRE11-dependent resection of hydroxyurea (HU)—stalled replication forks (2). This resection of nascent DNA occurred due to the inability of these cells to form stable RAD51 nucleofilaments, thereby resulting in a diminished ability of RAD51 to protect stalled replication forks. Subsequent studies established the role of the other core HR effector BRCA1 as well as the FA pathway components FANCA and FANCD2 as serving critical functions in RAD51 stabilization on nascent DNA, hence protecting stalled forks from degradation (3). These initial studies also established chromosomal instability as a direct consequence of fork degradation (2,3), therefore identifying a major mechanism of genome instability and cancer predisposition associated with mutations in the BRCA and FA genes. In addition to this, the well appreciated role of BRCA1 and BRCA2 mutations in cancer predisposition has led to the BRCA pathway being most widely studied in the context of fork protection, with RAD51 as the primary effector. Indeed, perturbing RAD51 activity in WT cells through the ectopic expression of the BRC4 peptide (which sequesters RAD51 from chromatin) or of RADX (which competes with RAD51 for ssDNA) renders forks susceptible to degradation (2–4). Similar effects were observed upon treatment with B02, a small molecule inhibitor of RAD51 (5).

A critical enabler of excessive fork degradation in cells lacking a functional FA/BRCA-pathway is fork reversal. However, the potential role of fork reversal in the resection of stalled forks was first established in the context of BRCA-proficient cells subjected to HU-mediated fork stalling. Specifically, cells subjected to prolonged HU treatment were found to exhibit fork degradation through an alternative pathway involving the nuclease DNA2 and the WRN helicase (6). Importantly, this degradation was enhanced upon the depletion of RECQ1, a specialized helicase responsible for restarting reversed replication forks. Moreover, depletion of RAD51 rescued this degradation, in line with its previously established role in mediating fork slowing and reversal upon exposure to replication stress (7). Subsequent work in BRCA-deficient systems showed that the SNF2 family translocases SMARCAL1, ZRANB3 and HLTF, which remodel replication forks into reversed fork substrates, are essential for the degradation of stalled forks (5,8,9). Intriguingly, studies in BRCA-deficient cells also shed light on the surprising dichotomous roles of RAD51 in both protecting stalled replication forks upon formation of stable nucleofilaments, as well as promoting their degradation by orchestrating fork reversal (9). These opposing roles of RAD51 were further exemplified through gradients of RAD51 depletion using titrated RNA interference in BRCA-proficient cells, where partial RAD51 depletion promoted fork degradation but complete depletion rescued fork stability (4).

The study of fork reversal in BRCA2-deficient cells also provided valuable insights into the mechanisms underlying the pathological consequences of fork degradation. Specifically, in BRCA2-deficient cells, the degradation of reversed forks prompts a controlled induction of double strand breaks (DSBs) by MUS81, triggering a POLD3-mediated fork restart—highly reminiscent of the break induced replication (BIR) pathway of fork rescue (10). Indeed, it was shown that RAD52 promotes MRE11-mediated degradation, setting the stage for fork cleavage, in line with its role in orchestrating BIR—dependent DNA synthesis, likely during mitosis (11,12). However, it is still unclear whether BIR contributes to chromosomal aberrations in cells lacking BRCA1 or FA pathway function.

## EMERGING DETERMINANTS OF FORK PROTECTION

### RAD51-dependent fork protectors

Recent studies have revealed the existence of several previously unknown factors involved in the protection of stalled forks from degradation. Interestingly, these factors employ fork protection mechanisms often distinct from the FA/BRCA pathway. However, several of these mechanisms still rely on the fork protection activity of RAD51. A relatively well-studied enabler of RAD51-mediated fork protection is BOD1L, which operates in the FA pathway of genome stability (13). However, unlike the mechanism previously described for the FA pathway (3), loss of BOD1L promotes fork degradation in a manner dependent on DNA2 rather than MRE11. Despite this, these observations remain in line with the role of excessive nuclease-mediated resection as underlying genome instability in FA cells (14). Further studies have revealed that the fork protection function of BOD1L occurs through its interaction with the histone methyltransferase SETD1A which triggers the histone chaperone function of FANCD2 to mediate RAD51 recruitment at stalled forks, in addition to inhibiting the pro-resection activity of CHD4 (15). These functions of BOD1L-SETD1A likely explain the role of FANCD2 in protecting against fork degradation in BRCA-deficient cells (16,17). Another recently described fork protection factor operating through RAD51 is WRNIP1 (18). Specifically, WRNIP1 was shown to mediate both replication fork restart as well as protection through its distinct ATPase activity and RAD51 stabilization functions, respectively. Interestingly, WRNIP1 depletion results in MRE11-dependent fork degradation and does not enhance fork degradation in BRCA2 depleted cells, suggesting a potential epistasis through RAD51 stabilization. Despite protecting against two different nucleolytic degradation pathways, both BOD1L and WRNIP1 interact with BRCA2, implying their potential roles as effectors in BRCA-mediated fork protection (13,18).

A unique player in the RAD51-mediated fork protection pathway is RAD52. Despite its role in promoting MRE11-mediated degradation in BRCA2-deficient cells (9), recent studies revealed that inactivation of RAD52 itself, in an otherwise BRCA-proficient background, predisposes forks to MRE11-mediated degradation (19). This fork protection function is unrelated to any RAD51-stabilizing activity of RAD52. Rather, RAD52 likely functions to safeguard RAD51 pools in cells upon induction of replication stress by counteracting the fork reversal function of SMARCAL1. Moreover, this function appears to operate independently of the RAD52-MUS81 pathway of fork rescue.

### RAD51-independent fork protectors

The occurrence of fork degradation independently of defects in a RAD51-mediated fork protection mechanism was documented in HR-proficient cells subjected to prolonged HU-mediated fork stalling (6). As mentioned in previous sections, prolonged stalling triggers fork degradation through the alternative DNA2-WRN-associated pathway. This degradation is further exacerbated by inactivating the helicase RECQ1, which functions in resolving reversed fork intermediates. Importantly, in later studies, using RADX depletion to ameliorate potential defects in RAD51 function failed to prevent fork degradation in this setting (4). Functional evidence suggests that this DNA2-mediated degradation aids in the restart of stalled/reversed replication forks. Indeed, these findings appear to be in line with the previously described role of DNA2 in suppressing the prevalence of reversed forks in fission yeast, thereby presumably preventing fork collapse (20).

Interestingly, recent work has revealed novel players which operate specifically in protecting replication forks from undergoing degradation through the DNA2-WRN pathway. One such fork protection factor is ABRO1, a paralog of ABRAXAS, a BRCA1-interacting protein (21). ABRO1-mediated fork protection occurs independently of RAD51-mediated protection. Interestingly, the inability of ABRO1-deficient cells to protect forks from the DNA2-WRN pathway of degradation correlates with pathological defects in genome maintenance, such as accumulation of mitotic defects and 53BP1 nuclear bodies.

We recently uncovered an unexpected role for PCNA ubiquitination in protecting stalled replication forks from degradation through the DNA2-WRN pathway (22). In line with the apparent mutual exclusivity of DNA2-WRN dependent fork degradation pathways and defects in RAD51-mediated fork protection, we found that suppression of fork degradation by PCNA ubiquitination does not involve RAD51 activity. Rather, defects in fork protection in cells unable to ubiquitinate PCNA at the K164 residue (PCNA-K164R cells) were associated with previously described defects in PCNA unloading from the lagging strand, caused by defective Okazaki fragment ligation (23) resulting from the inability to mitigate replication-associated gaps (22). In accordance with previous literature (24,25), the inability to unload PCNA from the lagging strand coincided with a loss of CAF-1 function in replication-associated nucleosome assembly, presumably caused by its abnormal sequestration through aberrant PCNA interactions. Indeed, we found that independent inactivation of factors governing each step, including: PCNA ubiquitination, LIG1 (Okazaki fragment ligation), ATAD5 (PCNA unloading) and CAF-1, triggered DNA2-driven fork degradation. Similar to ABRO1, the BRCA-independent nature of the PCNA ubiquitination-dependent fork protection was confirmed by the finding that loss of PCNA ubiquitination further exacerbated fork degradation in BRCA2-depleted cells. Like ABRO1, the failure to protect forks upon loss of PCNA ubiquitination was associated with pathological consequences such as HU-induced DSBs and the accumulation of 53BP1 nuclear foci.

Recent work has revealed roles for AND1 and TIM1, members of the fork protection complex (FPC, composed of TIM1, TIPIN, CLASPIN and AND1) in protecting stalled forks from MRE11-mediated degradation. A role for TIM1 in fork protection was identified through the characterization of the PCNA-interacting genome surveillance protein SDE2 and its function in preventing fork degradation (26,27). Specifically, SDE2 was found to promote the association of TIM1 with replication forks, ensuring their protection from MRE11-mediated resection upon stalling. Similarly, AND1 was found to protect replication forks from MRE11-mediated degradation (28). Importantly, it was observed that ssDNA at digested fork substrates efficiently recruited RAD51 upon AND1 ablation, suggesting that RAD51 mediates fork protection independently of AND1 - and by extension the FPC.

A unique fork integrity pathway depends on the Y-family polymerase POLK. Recent studies showed that inactivation of POLK results in MRE11-dependent degradation of stalled forks (29). Notably, the ability of POLK to protect stalled forks appears to be linked to its role in restarting stalled replication forks in a manner dependent on the FA pathway. Furthermore, in line with known roles of Y-family polymerases in binding to ubiquitinated PCNA in order to mediate DNA damage tolerance (30,31), the ubiquitin binding domain (UBD) of POLK is essential for fork restart. However, it remains unclear whether the interactions of POLK with ubiquitinated PCNA entirely account for its fork protection function since, as mentioned above, PCNA ubiquitination operates in a distinct fork stability pathway, which restricts degradation by DNA2-WRN (22). Furthermore, whether the fork protection function of POLK depends on RAD51 stabilization, as seen with the FA/BRCA pathway, remains unclear. Aside from POLK, other Y-family polymerases have also shown to potentially help bolster fork integrity. Recent work revealed a dependence of FA mutant cells, which are hypersensitive to interstrand crosslink (ICL)-inducing agents, on POLI for fork protection and restart upon stalling (32). Roles for POLN and POLK in promoting crosslink repair have also been reported (32,33), suggesting a possible general function of Y-family polymerases in mitigating ICL-induced replication stress.

Components of the non-homologous end joining (NHEJ) pathway of DSB repair have also been implicated in protecting the stability of stalled replication forks. Recent works have revealed a role for the NHEJ effector RIF1 in protection from the DNA2-WRN pathway of degradation (34,35). Notably, this fork protection function occurs independently of 53BP1, but instead depends on interactions of the C-terminal domain of RIF1 with protein phosphatase 1 (PP1). DNA2-mediated degradation in RIF1-deficient cells was found to promote genome instability and sensitivity to replication stress, owing to defects in replication fork restart. Interestingly, in these studies, both DNA2 and WRN were found to be hyperphosphorylated in the absence of RIF1. However, if this hyperphosphorylation alters the function of DNA2-WRN at replication forks is unclear.

Despite the well-documented role of the loss of 53BP1, a master regulator of NHEJ, in restoring HR in BRCA1-deficient cells, 53BP1 loss has not been previously reported to exhibit functions in fork protection (36,37). Interestingly, in addition to promoting BRCA1-RAD51 recruitment at DSBs, the 53BP1 interactor TPX2 was recently shown to function in protecting stalled replication forks (38). This activity of TPX2 occurs in a manner parallel to the BRCA1 fork protection pathway and through its interaction with Aurora A. However, unlike in BRCA1-deficient cells, loss of 53BP1 ameliorates fork stability in TPX2-deficient cells, revealing a previously unappreciated function of 53BP1 in fork protection. Moreover, recent work also revealed an unexpected role of 53BP1 in suppressing DNA2-mediated nascent strand degradation in BRCA-proficient cells (39). Interestingly, this role of 53BP1 in replication fork protection was found to not be universally conserved, but rather stochastically dependent on cellular context and the nature of 53BP1 inactivation. These findings potentially reconcile the previously contrasting observations regarding the role of 53BP1 in maintaining replication fork stability (5,38,40,41).

Recently, the Ku complex was found to mediate fork protection in *Schizosaccharomyces pombe* (42). It was shown that upon binding DNA ends of reversed replication forks, Ku suppresses extensive resection of these structures. Upon fork reversal, the removal of Ku by the MRN-Ctp1 complex acts as a rate-limiting step prior to long range resection by EXO1. Intriguingly, the nuclease activity of MRE11 was found to be dispensable for the removal of Ku. This is in contrast to previous reports of Ctp1 homologs Sae2 (*S. cerevisiae*) and CtIP (human) functioning to trigger the endonuclease activity of MRE11 at protein-blocked 5′ DNA ends (43,44). In light of the well-documented role of the MRE11 nuclease activity in resecting forks, these observations suggest a differential regulation of MRE11 at forks without RAD51 protection defects, or perhaps simply a differential regulation of MRE11 at stalled forks in fission yeast.

Despite its role in the initiation of fork resection, loss of CtIP was recently found to result in DNA2-mediated degradation of stalled forks (45). While this activity appears to depend on its nuclease activity, CtIP-mediated fork protection was found to operate in the same pathway as BOD1L. Furthermore, loss of CtIP showed synergy with loss of BRCA1 in fork degradation and compromised the survival of BRCA1-deficient cells. Epistasis of CtIP with BOD1L in fork protection implies a possible indirect role of CtIP in contributing to RAD51-mediated stabilization (13,15). Indeed, recent work characterizing loss-of-function CtIP mutations found in individuals with high breast cancer risk, revealed a function for CtIP in stabilizing RAD51 at replication forks, thereby protecting them from degradation (46). The study of these mutations revealed that the fork-protective activity of CtIP depends on its Sae2-like domain and works by antagonizing the anti-recombinase activity of FBH1. Similar to CtIP, the MRE11-interacting protein EXD2, was also revealed as a guardian of fork stability. Through its nuclease activity, EXD2 prevents the accumulation of reversed forks which may otherwise be degraded (47). Similar to CtIP, which contributes to the survival of BRCA1-deficient cells, EXD2 is also required for cellular fitness in both BRCA1 and BRCA2 deficient cells.

### Emerging insights into fork reversal pathways in fork degradation

As mentioned in previous sections, fork reversal is an important prerequisite for nuclease-mediated degradation of replication forks. In BRCA-deficient cells, fork reversal underlying fork degradation is RAD51-dependent. Importantly, RAD51 paralogs, namely RAD51B, RAD51C, RAD51D, XRCC2 and XRCC3, have also been revealed to be critical modulators of RAD51-mediated fork reversal (48). Specifically, the complex comprised of RAD51B,C,D and XRCC2 was found to be essential for fork slowing and reversal, thereby priming them for degradation in BRCA-deficient settings.

**Figure 1.**
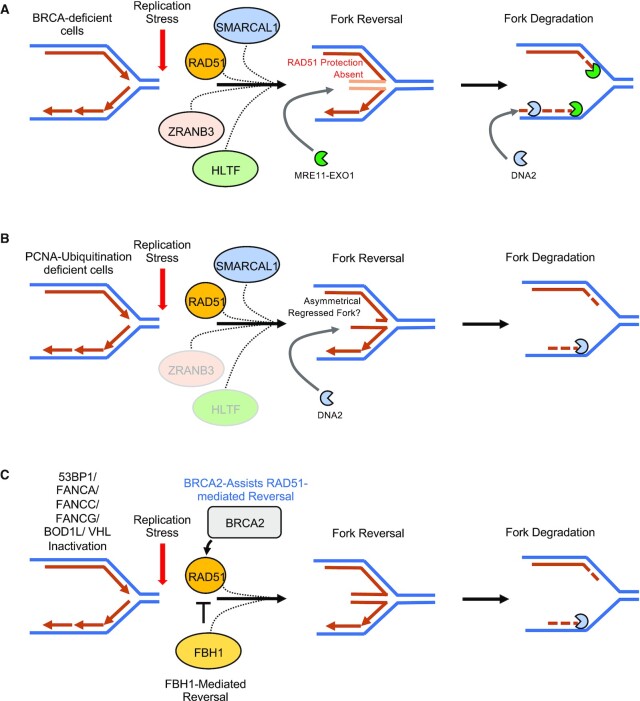
Fork reversal factors and their impact on fork degradation. (**A**) Fork degradation in the context of BRCA-deficiency requires a coordinated function of the SNF2-family translocases SMARCAL1, ZRANB3 and HLTF, in addition to RAD51, in order to mediate fork reversal. Inactivation of either of these translocases is sufficient to rescue fork stability in BRCA-deficient cells. (**B**) Fork degradation in the context of PCNA ubiquitination primarily involves the fork reversal activity of SMARCAL1, whereas ZRANB3 and HLTF show reduced roles. (**C**) Fork reversal and subsequent degradation in the context of 53BP1, FANCA, FANCC, FANCG, BOD1L or VHL inactivation depends on the helicase FBH1. Interestingly, in this context, BRCA2 aids in FBH1-mediated fork degradation by putatively bolstering RAD51 function against the RAD51-inhibitory effects of FBH1.

RAD51-dependent fork reversal is typically catalyzed by the SNF2-family DNA translocases SMARCAL1, ZRANB3 and HLTF (5,8–10). Interestingly, despite having distinct fork substrate preferences (49,50), depletion of each of the individual fork remodelers SMARCAL1, HLTF and ZRANB3 results in a complete rescue of fork stability in BRCA-deficient cells (5). This suggests that, at least in the context of BRCA deficiency, SMARCAL1, HLTF and ZRANB3 may act cooperatively to mediate fork reversal, with each translocase playing an essential role (Figure 1A). Interestingly, in cells unable to ubiquitinate PCNA, we uncovered that fork degradation showed no dependence on HLTF, a partial dependence on ZRANB3 and a complete dependence on SMARCAL1 (22) (Figure 1B). The partial dependence on ZRANB3 in this context is somewhat expected since PCNA polyubiquitination at the K164 residue enhances the interaction with PCNA and the fork slowing/reversal function of ZRANB3 (51,52). It remains unclear whether the lack of dependence on HLTF for fork reversal in PCNA ubiquitination-deficient cells reflects the role of HLTF in directly ubiquitinating PCNA (53,54), which may contribute to fork slowing by ZRANB3. HLTF also possesses an intrinsic translocase activity dependent on its HIRAN domain and readily remodels replication forks *in vitro*, as well as in cells upon the induction of replication stress (55–57). Overall, observations made in PCNA-ubiquitination deficient cells suggest that fork reversal may occur even without the coordinated activity of HLTF, ZRANB3 and SMARCAL1. Furthermore, each of these translocases have different fork substrates: SMARCAL1 shows a preference for leading strand-gaps, ZRANB3 for lagging strand gaps, and HLTF for unphosphorylated 3′-OH groups—potentially on leading strands or in the form of overhangs (49,50,57,58). Therefore, the selective activity of any of the translocases could potentially result in differences in the reversed fork structure. These differences may define the mechanism of fork degradation with regard to the nucleases involved (MRE11-EXO1 versus DNA2) as well as dependence on RAD51 nucleofilament formation for fork protection. For example, the MRE11 nuclease is able to utilize its endonuclease activity and subsequently catalyze 3′-5′ resection, enabling it to potentially act on both arms of reversed replication forks and prime the long range 5′-3′ resection activity of EXO1 (59,60). Therefore, it is possible that MRE11-mediated degradation is preferable in symmetrical reversed fork structures. In contrast, DNA2 is only able to catalyze 5′-3′ resection and is also known to show activity against 5′ ssDNA flaps arising during long-flap Okazaki fragment maturation (61–63). This raises the possibility of DNA2 requiring an asymmetrical reversed fork structure composed of an exposed 5′ ssDNA end in order to act as the sole nuclease responsible for resection. We therefore propose that in PCNA ubiquitination-deficient cells, the selective activity of SMARCAL1 in recognizing leading strand gaps, in combination with the relative inactivity of ZRANB3, results in a preponderance of reversed fork structures revealing a 5′ overhang. This 5′ overhang may be subsequently degraded by DNA2 independently of the initial endonuclease and 3′-5′ resection activity of MRE11 (Figure 1B).

Recently, fork degradation mechanisms which operate by catalyzing fork reversal independently of the SNF2-family translocases were uncovered (39). Specifically, cells deficient of FANCA, FANCC, FANCG, BOD1L, VHL or 53BP1, showed fork degradation which depended on RAD51 and the FBH1 helicase, previously found to catalyze fork reversal (64). Importantly, fork degradation in these contexts can occur independently of SMARCAL1, ZRANB3 and HLTF (Figure 1C). Furthermore, upon SMARCAL1, ZRANB3 and HLTF abrogation, FBH1-mediated fork reversal and degradation in a 53BP1-deficient background was found to be dependent on BRCA2. BRCA2 was also required for fork reversal and subsequent degradation upon inhibition of RAD51 with the specific inhibitor B02. These findings reveal a previously uncharacterized role of BRCA2 in catalyzing fork reversal, likely in the same pathway as FBH1 (Figure 1C). These observations are further complemented by the inability of BRCA-deficient cells to mediate efficient replication fork slowing upon exposure to low levels of replication stress (65). Conceptually, this could potentially underline the importance of BRCA2 in augmenting the function of RAD51 in restraining fork progression during replication stress. Additionally, the role of BRCA2 in mediating fork reversal could also offer a potential explanation for the dependence of BRCA-deficient cells on the concerted functions of SMARCAL1, ZRANB3 and HLTF in orchestrating fork reversal and subsequent degradation. Collectively, these findings reveal the existence of a number of fork remodeling pathways that directly influence the degradation of stalled replication forks.

While reversed forks appear to be the primary substrate for nucleolytic degradation, alternative fork substrates susceptible to nuclease activity have also been reported. The initial observation of MRE11-dependent post-replicative ssDNA gaps in RAD51-depleted *X. laevis* extracts, implies the existence of an entry point for MRE11 behind replication forks (1). Similar observations were later made in BRCA2-depleted *X. laevis* extracts (8). Importantly, while fork reversal contributes to extensive fork degradation, MRE11-dependent post-replicative gap expansion could also contribute to nascent DNA loss. A recent study investigating the effect of HU-induced metabolic imbalances on replication forks showed that accumulation of reactive oxygen species (ROS) triggers MRE11-dependent fork degradation as well gap accumulation (66). It was revealed that ROS specifically triggers ATM-dependent MRE11 phosphorylation, essential for the degradation of both stalled forks as well as post-replicative gap extension in progressing forks in HR-deficient cells. Since the absence of ROS precludes the apparent MRE11 activity at stalled forks, it is possible that the combined activity of MRE11 at reversed fork junctions as well as behind replication forks underlies HU-induced nascent DNA degradation. In this scenario, the absence of post-replicative gaps should preclude MRE11-dependent fork degradation. However, recent work identified separation-of-function BRCA2 mutants which rescued post-replicative gap formation but not fork protection (65). Overall, this suggests that while post-replicative gaps present a substrate for MRE11 activity, they may not be essential for the degradation of stalled forks, at least as measured by the DNA fiber assay. Nonetheless, it is possible that these gaps still play a role in enhancing nuclease-dependent fork instability. Further work will be needed to delineate their precise contribution.

### The emerging role of chromatin dynamics in fork protection

The importance of nucleosome positioning and chromatin context during DSB resection is well documented (67,68). This raises the question of whether nucleosome dynamics and chromatin states also affect the susceptibility of stalled replication forks to nucleolytic degradation.

**Figure 2.**
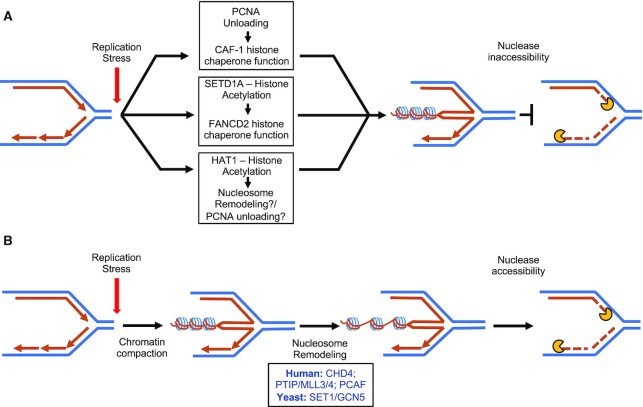
The impact of histone modifications and nucleosome remodeling on replication fork stability. (**A**) The ability to mediate efficient histone chaperone activity by CAF-1 and FANCD2 is associated with replication fork protection. The chaperone activity of CAF-1 is enabled by efficient PCNA-unloading from the lagging strand, which in turn depends on Okazaki fragment ligation and efficient lagging strand gap-filling by ubiquitinated PCNA. The fork protective histone chaperone activity of FANCD2 depends on the histone methyltransferase activity of SETD1A, which in turn restricts the activity of the CHD4, a member of the repressive NuRD complex. HAT1 promotes replication fork protection putatively through histone acetylation as well as ensuring timely PCNA unloading. (**B**) Nucleosome remodeling may contribute to fork degradation by enhancing the activity of nucleases to reversed fork substrates. CHD4 enables the resection of stalled replication forks in cells deficient in BRCA2 and FANCD2, by promoting the activity of MRE11. The methyltransferase activity of the PTIP interacting histone methyltransferases MLL3/4 promote fork resection in BRCA-deficient cells by enabling the recruitment of MRE11. Similarly, the H4K8 acetyltransferase activity of PCAF promotes the recruitment of MRE11/EXO1 to stalled forks in BRCA-deficient cells. Finally, in yeast models, the methyltransferase and acetyltransferase activities of SET1 and GCN5 respectively are required for nucleosome remodeling events enabling the MRX complex to resect DNA at stalled replication forks.

The presence of nucleosomes in double stranded DNA directly counteracts long range nucleolytic resection (69). In recent work, we demonstrated that perturbing replication-associated nucleosome deposition by inactivating chromatin assembly factor-1 (CAF-1), predisposes stalled forks to DNA2-dependent degradation (Figure 2A) (22). Indeed, previous research in yeast delineated the role of CAF-1 in establishing nucleosome periodicity, which closely correlates with Okazaki fragment length (70,71). Importantly, in the context of PCNA ubiquitination deficiency, where the inability to unload PCNA from lagging strands putatively sequesters CAF-1 away from active replication forks, the stability of reversed forks is compromised (22). This suggests a function of CAF-1 in stabilizing reversed replication forks. The role of nucleosomes in protecting reversed forks was elucidated in further detail in RNF168-depleted systems, wherein histone H2A ubiquitination was found to be essential for fork progression, stability and restart (41). Through psoralen-crosslinking coupled with EM under denaturing conditions, reversed forks were found to be increased in RNF168-depleted cells and to contain ssDNA bubbles consistent with standard nucleosome periodicity. These observations indicate that nucleosomes can assemble on reversed forks and influence replication fork dynamics.

Chromatin remodelers were found to be important in determining the replication-coupled periodicity of nucleosomes (71) and influence DNA resection (67,68), suggesting their potential role in impacting fork stability. Interestingly, in yeast, the MRE11-RAD50-XRS2 (MRX) complex (equivalent to the MRN complex in metazoans), was shown to directly participate in chromatin remodeling to facilitate resection at stalled replication forks (72). Specifically, MRX-associated remodeling depends directly on the activity of the yeast chromatin remodelers CHD1, ISW1 and the RSC complex, which in turn rely on the H3K4 methyltransferase and acetyltransferase activities of SET1 and GCN5 respectively, enabling increased chromatin accessibility (Figure 2B). Similarly, in BRCA-deficient cells, the H3K4 and H4K8 methyltransferase activities of MLL3/4 and PCAF, respectively, promote the resection of stalled replication forks (Figure 2B) (37,73).

Interestingly, histone modifications that increase chromatin accessibility and subsequent remodeling do not exclusively work to facilitate fork resection but rather can also promote fork protection. For example, a dependence on the H3K4 methyltransferase SETD1A for fork protection was observed in mammalian cells (15). Here, the requirement of H3K4 methylation was found to restrict the activity of CHD4, an ATPase component of the repressive NuRD complex, and promote the histone chaperone activity of FANCD2, thereby assisting in fork stability (Figure 2A). Conversely, depletion of CHD4 was shown to restore fork protection in BRCA2-deficient cells by suppressing the recruitment of MRE11 at HU-stalled forks (Figure 2B) (37). Similarly, the loss of the histone acetyl transferase HAT1, primarily associated with increased chromatin accessibility, also renders stalled forks susceptible to degradation (Figure 2A) (74). Another fork protection factor putatively dependent on chromatin accessibility is FANCJ. Notably, loss of FANCJ is associated with both fork degradation as well as abnormal chromatin compaction via H3K9-trimethylation (H3K9me3) at nascent DNA upon treatment with HU (75,76). However, it remains unclear whether excessive chromatin compaction at stressed nascent DNA in FANCJ-deficient cells directly underlies fork degradation or simply reflects a protective mechanism against excessive fork resection. Recent work in fission yeast implies that *de novo* chromatin compaction at replication forks during replication stress may indeed confer protection against fork instability (77). Specifically, replication stress was shown to trigger H2BK33-deacetylation and H3K9me3 accumulation at replication forks. Importantly, H2BK33-deacetylation was found to prevent the untimely uncoupling of replisome components, thereby aiding fork restart and protecting against fork collapse.

Collectively, these observations underline the importance and the context-dependency of histone modifications in directly enabling the activities of chromatin remodelers and histone chaperones, thereby defining the nucleosomal landscape for the resection of stalled forks.

## FORK PROTECTION AND THE IMPACT ON CELLULAR SURVIVAL

### Restoration of fork protection

Recent studies identified an association between the restoration of fork stability and chemoresistance in BRCA-deficient cells, making fork stability an important component of the etiology of BRCA-mutant cancers. (5,37). As mentioned in earlier sections, a fundamental determinant of replication fork degradation is fork reversal mediated by the SNF2 family DNA2 translocases SMARCAL1, ZRANB3 and HLTF (5,8–10). Perturbing the activities of each of these fork remodelers restored not only fork protection but also resistance to cisplatin and PARP inhibitors (PARPi) in BRCA1-deficient cells (5). However, the impact of abolishing fork reversal through translocase inactivation on genome stability in BRCA-deficient cells is less clear. While abolishing fork reversal by SMARCAL1 and ZRANB3 depletion rescued DSB formation and chromosomal aberrations in BRCA1/2-depleted cells treated with camptothecin (CPT) (5), ZRANB3 loss in BRCA2-depleted cells was found to further exacerbate chromosomal abnormalities upon treatment with HU (9). These divergent effects can be possibly explained by the differences in how HU and CPT create replication stress, thereby resulting in varied outcomes upon abolishing fork reversal in BRCA-deficient cells. Further work is needed to precisely reconcile these differences.

As mentioned in the previous section, loss of CHD4 restores both chemoresistance and fork protection in BRCA2-deficient cells (37,78). A possible explanation for this is the function of CHD4 as a chromatin remodeler, acting in in a manner opposing SETD1A-mediated H3K4 methylation thereby facilitating resection (15). However, previous work has suggested that the mechanism of chemoresistance upon CHD4 depletion in BRCA2-deficient cells is through the enhancement of RAD18-mediated PCNA ubiquitination (78). Since loss of PCNA ubiquitination enhances fork degradation in BRCA2-depleted cells (22), it is plausible that PCNA ubiquitination triggered by CHD4 loss could bolster fork protection in BRCA2-deficient cells. However, loss of CHD4 in BRCA2-deficient cells may also contribute to chemoresistance independently of fork protection, by augmenting PCNA ubiquitination-dependent translesion synthesis (TLS). Similar to the loss of CHD4, inactivation of the NHEJ component PTIP and of the PTIP-interacting H3K4 methyltransferases MLL3/4 also rescues fork stability and confers chemoresistance to BRCA2-deficient cells (37).

Restoration of RAD51 function was recently shown to rescue fork stability in cellular backgrounds which fail to form stable RAD51 nucleofilaments at stressed replication forks. Specifically, the loss of RADX, a competitor of RAD51 in binding to ssDNA, restores fork protection in cells deficient in components of the FA/BRCA pathway (4,79). Increasing RAD51 binding to DNA by perturbing RADX restores fork protection independently of restoring the function of upstream FA/BRCA pathway components. On similar lines, in recent work we uncovered that loss of E2F7, a transcriptional repressor of RAD51, restores RAD51 recruitment to chromatin, thereby promoting fork stability in BRCA2-deficient cells (80).

Through unbiased CRISPR knockout screens, we also established that loss of the acetyltransferase TIP60, previously described as a suppressor of 53BP1 (81,82), confers resistance to the PARPi olaparib, and restores replication fork protection to BRCA2-deficient cells (83). Importantly, the olaparib resistance upon TIP60 depletion was dependent on the NHEJ effectors 53BP1 and REV7, and involved suppression of resection at olaparib-induced DSBs. However, it is unclear whether the restoration of fork stability in BRCA2-deficient cells upon TIP60-depletion also arises as a result of suppression of DNA resection, dependent on NHEJ components.

### Fork protection and cell survival

Restoration of fork protection is associated with acquired chemoresistance (5,37). However, much of recent work suggests that the restoration of fork protection may not always translate to enhanced cell survival. Rather, this may depend on the context of the genetic background as well as the nature of replication stress encountered.

In the initial characterization of the function of BRCA2 in protecting stalled replication forks, it was found that HU-induced fork degradation did not directly translate into HU sensitivity (2). This is in contrast to the role of fork protection in restoring chemoresistance to both BRCA1- and BRCA2-deficient cells (37). Unlike the loss of BRCA2 function, loss of BRCA1 was found to confer sensitivity to HU (84). This fork protection function occurs independently of the BRCA1–PALB2 interaction (and therefore its interaction with BRCA2), but rather depends on the interaction of BRCA1 with BARD1 (84,85). This mutual independence of BRCA1 and BRCA2 in their fork protection activities might, in part, explain the observed differential impact of fork degradation on cellular survival. Inactivation of fork reversal has only been documented to restore chemoresistance to BRCA1-deficient cells (5), suggesting a differential impact of SMARCAL1, HLTF and ZRANB3 inactivation in the context of either BRCA1 or BRCA2 deficiency on DNA damage accumulation and repair. Interestingly, BRCA1 deficient cells were shown to mount an adaptive response to cisplatin pre-treatment by upregulating PRIMPOL-mediated repriming, thereby counteracting fork reversal and degradation and ensuring cell survival (86).

Fork protection defects are also a hallmark of FA pathway deficiency (3,87). In the absence of FANCD2, aberrant activity of DNA2 was found to underlie defective ICL repair (14), suggesting a role for FA-mediated fork protection at ICL-stalled forks. In line with this, inactivation of fork protection factors such as BOD1L-SETD1A, which operate in the same pathway as FANCD2 in preventing DNA2-mediated degradation of forks, underlies sensitivity to ICL-inducing agents (13,15). Similarly, RAD51 mutations identified in individuals with FA-like presentation were shown to cause fork degradation and ICL sensitivity (88). Importantly, recent work also revealed that BRCA2 DNA-binding domain (DBD) mutants engender FA-like presentations, sensitivity to ICLs via excessive DNA2-mediated resection and fork protection defects (89). Intriguingly, unlike what was observed for BRCA1 (5), fork reversal through SMARCAL1, HLTF and ZRANB3 did not play a role in ICL-induced fork resection in these BRCA2 DBD-mutant cells. Conceptually, these findings indicate overlapping functions of the BRCA pathway with the FA pathway of ICL repair and highlight the importance of BRCA2-RAD51-mediated fork protection in cellular survival upon treatment with ICL-inducing agents.

Recent evidence has linked the restoration of fork protection to synthetic viability in BRCA-deficient backgrounds. Knockdown or genetic ablation of PARP1 was shown to restore viability to mouse embryonic stem cells (mESCs) bearing a homozygous genetic knockout of BRCA2 (BRCA2-KO) (90). Importantly, it was found that loss of PARP1 had no effect in restoring HR but rather restored fork protection to these cells. Similarly, loss of either PTIP or RADX, which promotes fork stability in BRCA-deficient cells, also restores synthetic viability to BRCA2-KO mESCs, without rescuing HR (37,79).

In line with the role of fork protection in restoring viability, the further enhancement of fork degradation was shown to contribute to synthetic lethality in BRCA-deficient cells. BRCA-deficient cells were found to be hyperdependent on FANCD2 for fork protection and survival (16,17). However, FANCD2 was also found to enhance the recruitment of POLQ to damaged DNA, thereby promoting alternative end-joining (17), in line with a role for POLQ in enhancing the survival of BRCA-deficient cells. Indeed, BRCA-deficient tumors were found to be dependent on POLQ-mediated alternative-NHEJ for survival (91,92). Loss of CTIP function, which underlies fork degradation, also synergizes with the loss of BRCA1, resulting in a further exacerbation of fork degradation and synthetic sickness in BRCA1-deficient cells (45). Similar to FANCD2, the deubiquitinase USP1 was found to be required for fork protection in BRCA1 deficient cells and loss of USP1 triggered lethality in these cells (93). Finally, loss of PRIMPOL results in fork degradation and exacerbates the growth defects of BRCA1-mutant cells (86). Collectively, these observations highlight the importance of fork protection in determining cellular fitness in the context of BRCA deficiency.

### Fork stability and PARPi sensitivity

**Figure 3.**
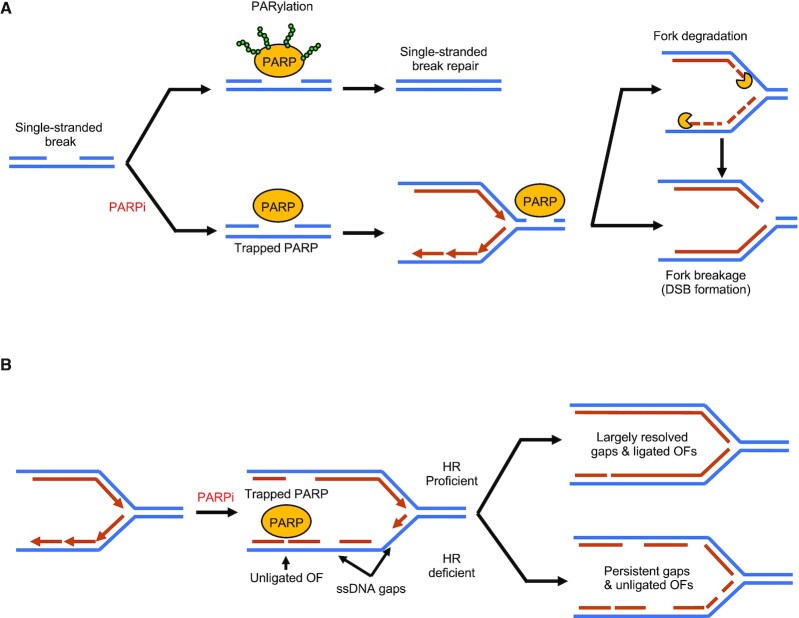
Proposed mechanisms of PARPi-mediated cellular lethality in BRCA-deficient cells. (**A**) The original models proposed fork degradation and DSB-induction as major mechanisms contributing to PARPi-mediated synthetic lethality in BRCA-deficient cells. Specifically, PARPi-induced trapping of PARP1/2 on the chromatin poses obstacles to replication fork progression thereby causing replication stress which putatively results in fork degradation and/or fork cleavage. (**B**) A proposed alternative model suggests ssDNA gap induction as the major mechanism underlying PARPi-mediated cellular lethality. In HR-deficient cells, PARPi can potentially create persistent ssDNA gaps by inhibiting timely fork slowing in the face of replication stress as well as interfering with the resolution of ribonucleotide excision intermediates owing to PARP-trapping. PARPi can also interfere with the resolution of Okazaki fragment ligation by inhibiting lagging strand gap-filling and cause the potential accumulation of trapped PARP at unligated Okazaki fragment intermediates. A combination of persistent ssDNA gaps and unligated Okazaki fragments may lead to cellular lethality in HR-deficient cells.

Restoration of fork stability often correlates with resistance to PARPi (Figure 3A). Restoration of fork stability by abrogating fork reversal was shown to ameliorate PARPi sensitivity in BRCA1-deficient cells (5). Similarly, RADX depletion, which restores fork protection in BRCA-deficient cells without rescuing HR, also restores PARPi resistance in BRCA2-deficient cells (79). Furthermore, treatment with olaparib in concert with HU further enhances fork degradation in BRCA2-deficient cells (94). The amelioration of PARPi sensitivity upon restoration of fork protection appears in many cases to be context-dependent. For example, abrogation of fork reversal has not been documented to improve survival upon PARPi treatment in BRCA2-deficient cells. On similar lines, RADX inactivation, while restoring fork protection to BRCA1-deficient cells, does not impact the survival of these cells upon treatment with PARPi (4). These observations indicate that conventional fork protection, while a contributing factor, may not be the primary determinant of PARPi sensitivity.

A specific context in which PARPi sensitivity correlates with fork protection defects is that of the activation of ssDNA-induced innate immune response. Nucleolytic processing of stalled replication forks was shown to induce activation of the cGAS-STING pathway triggered by the presence of cytosolic ssDNA (95). Furthermore, PARPi treatment was shown to trigger the expression of interferon-stimulated genes and activation of the cGAS-STING pathway in BRCA2-deficient cells, owing to the presence of cytosolic DNA (96). Similarly, in mouse models of BRCA1-deficient ovarian cancer, the cGAS-STING pathway was found to be critical for PARPi-dependent reduction of tumor size (97). It is therefore possible that, in the context of an intact innate immune signaling, restored fork protection becomes an important component of acquired PARPi resistance. Indeed, in orthotopic transplantation mouse models, acquired PARPi resistance of BRCA2-deficient tumors was found to coincide with restoration of fork stability (37). This suggests that fork protection is a potential mechanism by which tumors avoid immune surveillance and become resistant to PARPi *in vivo*.

A significant body of work identified restoration of DSB repair as a major mechanism of acquired PARPi resistance. Our recent work showed that loss of E2F7 promotes PARPi resistance in BRCA2-deficient cells, and this correlates with both fork protection and HR restoration (80). BRCA2 DBD mutants that display fork protection defects (likely explaining their MMC sensitivity) also show sensitivity to PARPi (89). While moderately compromised, HR was not abolished in these cells. Moreover, we recently showed that loss of the acetyltranferase TIP60 confers PARPi resistance to BRCA2-deficient cells through the restoration of accurate end-joining DSB repair (83). In BRCA1-deficient cells, loss of 53BP1 or of the Shieldin complex, which is a critical effector of 53BP1-mediated NHEJ, restores HR and PARPi resistance (36,98–104). Collectively, these findings underline the importance of intact DSB repair as a major determinant of PARPi resistance (Figure 3A).

Another mechanism implicated in PARPi-mediated cytotoxicity is the trapping of PARP1 and PARP2 proteins on the chromatin. Trapped PARP1/2 could directly pose obstacles to DNA replication, and thereby potentially underlie fork degradation and DSB formation (Figure 3A,B). The cytotoxic effects of trapped-PARP lesions were first described in DT40 chicken lymphoblasts, where the genetic depletion of PARP1 was found to suppress PARPi sensitivity (105). Furthermore, recent studies revealed that loss of ALC1 drives PARPi sensitivity in HR-deficient cells by prohibiting the release of trapped PARP1 and PARP2 from chromatin (106–108). Genome-wide CRISPR screens have recently uncovered misincorporated ribonucleotides as a major source of PARP-trapping lesions (109). Specifically, TOP1-mediated cleavage of misincorporated ribonucleotides was shown to underlie PARPi sensitivity in RNASEH2-knockout cells, suggesting a role of TOP1 cleavage products in engaging and subsequently trapping PARP1. PARP-trapping was also shown to contribute to the transcriptional repression of p21 leading to an unbridled increase in fork speed, which could potentially exacerbate the effect of trapped-PARP lesions encountered by the replication fork (110).

We recently showed that PCNA-K164R cells, which have fork protection defects, are not sensitive to PARPi but instead exacerbate the PARPi sensitivity of BRCA-deficient cells (22). This further indicates that fork protection is unlikely to be a universal determinant of PARPi resistance. Instead, we found that lack of PCNA ubiquitination synergizes with BRCA deficiency in suppressing the accumulation of ssDNA gaps, which correlates with the synergistic sensitivity to PARPi. This raises the possibility that persistent ssDNA gaps, rather than defects in fork protection, are responsible for PARPi sensitivity. This is further corroborated by recent work showing that PARPi treatment results in formation of replication associated gaps, potentially exacerbating the predisposition to gap formation of BRCA-deficient cells (Figure 3B) (65,111). In these studies, PARPi resistance was found to be associated with increased capacity to suppress PARPi-induced gaps. Furthermore, restoration of gap suppression in the absence of fork protection was sufficient to confer PARPi resistance to BRCA2- or FA-deficient cells. The role of HR in suppressing ssDNA gaps is supported by the findings that RAD51 is required for post-replicative gap filling in response to bulky DNA lesions or the absence of translesion synthesis (1,112,113). However, the precise mechanism underlying the formation of PARPi-induced gaps remains unclear. One possibility could be related to the role of PARP1 in mediating fork slowing and reversal upon encountering replication stress (114). As BRCA-deficient cells are defective in fork slowing/reversal and gap-filling (22,37,65), unrestrained fork progression upon PARPi treatment could give rise to an accumulation of spontaneously occurring gaps resulting from endogenous replication stress in these cells. Another putative source of PARPi-induced ssDNA gaps are TOP1 cleavage products of misincorporated ribonucleotides (109), which could become persistent due to the inhibition of ssDNA gap repair by PARP1. In both situations, the persistence of newly acquired replication-associated gaps could become exacerbated in the absence of BRCA-mediated post-replicative repair. Indeed, studies in *X. laevis* egg extracts established a role for RAD51 and BRCA2 in suppressing gaps both at the junctions of replication forks, as well as MRE11-dependent gaps behind them (1,8), thus solidifying a *bona fide* connection between the BRCA/RAD51-mediated HR and novel gap evasion.

An unexpected role for PARP1 and PARP2 in ensuring the ligation of Okazaki fragments that escaped conventional ligation during S-phase DNA synthesis was recently revealed (115). Perturbation of Okazaki fragment maturation through chemical inhibition of FEN1 in PARP1/2-deficient cells was found to cause lethality. These observations suggest that an aberrant accumulation of unligated Okazaki fragments could itself be toxic to cells. Recent work including ours suggests that the inability to mitigate replication associated gaps, for example upon loss of PCNA ubiquitination, causes lagging strand synthesis defects through the preferential accumulation of lagging strand-associated gaps likely owing to frequent repriming by Polα (22,116,117). This indicates the possibility that, upon PARPi treatment, ssDNA gaps preferentially accumulate on the lagging strand in the absence of gap-filling pathways such as the BRCA pathway. Persistent lagging strand gaps undergoing delayed gap-filling in BRCA-deficient cells could later necessitate the engagement of PARP1/2 at unligated Okazaki fragment substrates. In this situation, PARPi could further exacerbate the prevalence of toxic unligated Okazaki fragments as well as lead to PARP-trapping lesions at these substrates, resulting in selective cytotoxicity in these cells (Figure 3B).

In conclusion, while fork protection may confer PARPi-resistance in certain backgrounds, it is possible that mechanisms which allow cells to evade catastrophic DSB-induction, PARP-trapping and ssDNA gap accumulation comprise the major channels by which PARPi resistance is restored.

## CONCLUDING REMARKS

In the past decade, replication fork degradation has emerged as a major mechanism underlying cancer-associated genome instability. Fork protection and its determinants have gained prominence in the context of BRCA deficiency. This is due to the fact that fork degradation observed in BRCA-deficient cells revealed fork protection as a major mechanism by which the BRCA pathway ensures genome stability. These findings are especially significant since they reveal a protective function of the BRCA pathway against replication stress, which is frequently encountered by cells undergoing DNA replication. Due to the relatively ubiquitous nature of replication stress, as opposed to DSBs—which represent the most severe form of DNA damage—replication fork protection can be regarded as a more clinically relevant mechanism by which genome stability is maintained by the BRCA pathway. In recent years, fork protection has also emerged as a major determinant cell survival and chemosensitivity, highlighting the inability to protect replication forks as a therapeutic vulnerability in BRCA-deficient cancers. Therefore, gaining a better understanding of the underpinnings of fork stability has important implications in the etiology of BRCA-deficient cancers as well as other cancers susceptible to fork degradation.

Recent efforts in characterizing fork protection defects have yielded not only a better understanding of how the BRCA pathway orchestrates fork protection, but also revealed a multitude of other factors which govern fork stability. Importantly, these newly emerging factors belong to pathways which either cooperate with the BRCA pathway or act independently of it in protecting replication forks. In this review, we examined these factors on the basis of their dependence on RAD51 to orchestrate fork protection, and the mechanisms of fork resection they protect against (MRE11, DNA2-WRN). We further examined the downstream effects of the loss of fork protection upon the inactivation of these pathways on genome stability, as well as the impact of their combined inactivation with the BRCA pathway on cell viability. We also explored the emerging role of nucleosome remodeling and chromatin dynamics as determinants of stalled fork resection. Importantly, histone modifications and nucleosome remodeling may have a direct impact on the accessibility of nucleases to stalled fork substrates, thereby influencing fork resection.

The inability to protect replication forks is associated with a vulnerability to treatment with chemotherapeutics in BRCA-deficient cells. Conversely, the artificial restoration of fork stability in BRCA-deficient cells has been shown to restore chemoresistance to these cells. This highlights the challenges in the treatment of BRCA-deficient cancers. In the last section, we examined mechanisms underlying the restoration of fork stability to BRCA-deficient cells. These include restoration of the RAD51 fork protective function, inactivation of replication fork reversal, increasing the reliance of BRCA-deficient cells on proteins which bolster fork stability (such as FANCD2, USP1 and PRIMPOL). Finally, we evaluated fork degradation as a mechanism contributing to PARPi-mediated synthetic lethality. Recent findings showed that fork degradation, though often associated with PARPi sensitivity, may not be universally causative of PARPi-mediated cellular lethality. Instead, a more significant correlation may be found between PARPi sensitivity and the inability to mitigate replication-associated ssDNA gaps. These gaps may arise as a result of the effect of PARPi in suppressing key functions of PARP1 in mediating replication fork slowing upon stress encounter, as well as inducing PARP-trapping as a result of the aborted resolution of misincorporated ribonucleotides during DNA replication. We suggest an importance of unligated Okazaki fragments as lesions potentially contributing to PARPi-mediated cellular lethality. Collectively, these observations imply a complex role of fork protection in determining progression as well as therapeutic outcomes in BRCA-mutant cancers. Furthermore, the breadth of the emergent determinants of fork stability discussed here, may indicate a more significant role of fork stability in cancer etiology than previously anticipated.
